# Investigating the Role of Myeloperoxidase and Angiopoietin-like Protein 6 in Obesity and Diabetes

**DOI:** 10.1038/s41598-020-63149-7

**Published:** 2020-04-10

**Authors:** Mohammad G. Qaddoumi, Muath Alanbaei, Maha M. Hammad, Irina Al Khairi, Preethi Cherian, Arshad Channanath, Thangavel Alphonse Thanaraj, Fahd Al-Mulla, Mohamed Abu-Farha, Jehad Abubaker

**Affiliations:** 1 Biochemistry and Molecular Biology, Dasman Diabetes Institute, Kuwait City, Kuwait; 2 Pharmacology and Therapeutics Department, Faculty of Pharmacy, Kuwait University, Kuwait City, Kuwait; 3 Department of Medicine, Faculty of Medicine, Kuwait University, Kuwait City, Kuwait; 4 Functional Genomic Unit, Dasman Diabetes Institute, 15462 Kuwait City, Kuwait

**Keywords:** Obesity, Type 2 diabetes

## Abstract

Myeloperoxidase (MPO) is positively associated with obesity and diet-induced insulin resistance. Angiopoietin-like protein 6 (ANGPTL6) regulates metabolic processes and counteract obesity through increased energy expenditure. This study aims to evaluate the plasma MPO and ANGPTL6 levels in obese and diabetic individuals as well as MPO association with biochemical markers of obesity. A total of 238 participants were enrolled, including 137 control and 101 type 2 diabetes (T2D) patients. ANGPTL6 and MPO levels and other biomarkers were measured via ELISA. ANGPTL6 levels were significantly higher in the diabetic population and obese individuals. When the group was stratified based on T2D, ANGPTL6 levels were significantly higher in obese-diabetic participants compared with non-obese-diabetics, but obese-non-diabetic individuals had similar ANGPTL6 levels to their controls. MPO levels were higher in obese compared with non-obese participants but did not differ between T2D and control participants. MPO levels were upregulated in obese compared with non-obese in both diabetics and non-diabetics. MPO was positively associated with ANGPTL6, triglyceride, BMI, TNF-alpha, high-sensitivity C-reactive protein, interleukin-6, and plasminogen activator inhibitor-1. Taken together, our findings suggest that both MPO and ANGPTL6 may regulate obesity, although MPO exerts this effect independent of diabetes while ANGPTL6 may have a modulatory role in diabetes.

## Introduction

Obesity is a major global health concern that affected over 650 million individuals worldwide in 2016^1^. At 18 years of age, individuals with body mass index (BMI) ≥ 30 kg/m^2^ have an increased lifetime risk of 55%–74% for diabetes^2^. In addition to the impact of obesity on the risk of diabetes, it is associated with an increased risk of cardiometabolic disorders, such as dyslipidemia, hypertension, heart failure, and atherosclerotic cardiovascular disease as well as decreased quality of life^3,4^.

Type 2 diabetes (T2D) occurs due to a progressive decline in the ability of the pancreas to secrete enough insulin as well as insulin resistance in insulin target tissues. The pathophysiology of T2D is characterized by excessive accumulation of ectopic fat in the liver, pancreas, and skeletal muscles, eventually manifesting as insulin resistance in these tissues and pancreatic beta cell dysfunction that ultimately leads to hyperglycemia^5^.

Obese mice have elevated plasma neutrophil levels^6^. Neutrophil infiltration into the white adipose tissue is an early event that promotes the development of diet-induced obesity and insulin resistance^7^. Similarly, patients who lose weight have decreased circulating neutrophil and monocyte levels^8^. As a result of chemokine production by neutrophils, macrophages are recruited into the adipose tissues where they secrete cytokines, such as tumor necrosis factor alpha (TNF-α) and interleukin-1 beta, causing obesity-mediated inflammation and impaired insulin signaling^9^. Myeloperoxidase (MPO) is the most abundant protein in human neutrophils, and it plays a major role in inflammation, oxidative stress, lipoprotein oxidation, and atherosclerosis^10–12^. Mice deficient in MPO are resistant to diet-induced obesity and insulin resistance, indicating that MPO may have a regulatory role in obesity^13^. Furthermore, the inhibition of MPO activity in the neutrophils via a non-specific peroxidase inhibitor decreased diet-induced insulin resistance in obese wild-type mice^14^. Obesity is one of the most common causes of insulin resistance. The main hallmark of obesity is chronic inflammation in the liver, skeletal muscles, and adipose tissues, which is otherwise known as insulin target tissues^15^. Moreover, MPO was associated with the increased risk of cardiovascular disease in prediabetic participants^16^. Furthermore, MPO and its reactive oxidants mediate the pathogenesis of numerous acute and chronic inflammatory conditions^17,18^.

Angiopoietin-like proteins (ANGPTLs) are a family of eight secreted glycoproteins with structural homology to angiopoietins, which are mainly involved in angiogenesis^19^. ANGPTL3, ANGPTL4, and ANGPTL8 are the most well-studied members of this family due to their important role in regulating lipid metabolism via inhibiting lipoprotein lipase activity^20–22^. Several ANGPTL proteins have been associated with obesity, insulin resistance, and diabetes^23–25^. ANGPTL6, also known as angiopoietin-related growth factor, is a liver-derived secreted protein that is initially involved in promoting angiogenesis, epidermal cell proliferation, and wound healing^26,27^. Animal knockout and transgenic studies have shown that ANGPTL6 counteracts diet-induced obesity and insulin resistance via increasing energy expenditure^28^. Furthermore, patients with T2D have elevated serum ANGPTL6 levels, and a positive association was observed between elevated serum ANGPTL6 levels and fasting blood glucose levels^29–31^. However, the role of both ANGPTL6 and MPO in both obesity and diabetes in humans has not been determined.

Thus, this study aimed to evaluate the plasma MPO and ANGPTL6 levels in both obese and T2D patients and to assess their association with the biochemical markers of obesity, inflammation, and atherosclerosis.

## Materials and Methods

### Study population and ethical approval

The study cohort comprised 238 participants, including 101 patients diagnosed with T2D and 137 participants without T2D. The participants were stratified according to BMI and were classified as either non-obese (19.5 ≤ BMI < 30 kg/m^2^) or obese (30 ≤ BMI < 40 kg/m^2^). Based on this stratification, the non-diabetic participants included 77 non-obese and 60 obese participants, and the T2D patients included 36 non-obese and 65 obese patients. The study was approved by the ethical review board of Dasman Diabetes Institute and was conducted in accordance with the Declaration of Helsinki. A written informed consent was obtained from all participants before the initiation of the study. The exclusion criteria were as follows: patients with type 1 diabetes, morbidly obese participants (BMI ≥ 40 kg/m^2^), those with a history of major illness or taking any medication and/or supplements known to influence body composition or bone mass, and those who engaged in any physical exercise within the last 6 months.

### Blood collection and biochemical measurements

Blood samples were collected from 238 study participants, and plasma was prepared using vacutainer EDTA tubes. Plasma samples were aliquoted and stored at −80 °C until assayed, as described previously^32^. Fasting plasma glucose, triglyceride, total cholesterol, low-density lipoprotein, and high-density lipoprotein were measured with Siemens Dimension RXL chemistry analyzer (Diamond Diagnostics, Holliston, MA). HbA1c levels were measured using the Variant device (Bio-Rad Laboratories, Hercules, CA). Hypertension and hyperlipidemia were self-reported by patients when they were asked if they suffered from other conditions.

### Plasma ANGPTL6 and MPO levels

The plasma ANGPTL6 and MPO levels were measured using enzyme-linked immunosorbent assay (ELISA) kits purchased from EIAab. The plasma samples were thawed on ice and centrifuged at 10,000 g for 5 min at 4 °C to remove any debris^32^. The ELISA kit was validated using recombinant ANGPTL6 and MPO at a known concentration in the plasma. A plasma dilution of 1:25 showed linearity and was used in the assay. The intra-assay coefficients of variation ranged from 7.5% to 9.2%, and the inter-assay coefficients of variation ranged from 7.9% to 9.6%.

### Plasma biochemical marker levels

The plasma leptin, adiponectin, and PAI-1 levels were measured using multiplexing immunobead array according to the manufacturer’s instructions (R&D Systems). The data were processed using the Bio-Plex Manager Software version 6 (Bio-Rad), with five parametric curve fitting. hsCRP was measured using ELISA, as previously reported^33^.

### Statistical analysis

Some of the continuous parameters measured in Tables 1–3 are normally distributed while some are not, therefore, data are expressed as Median (IQR) and Mann-Whitney U test was used to compare between the different groups. Otherwise, a student’s *t*-test was utilized and data are expressed as mean ± standard deviation (SD) when normally distributed. Pearson’s partial correlation coefficients were calculated to determine the association between MPO levels and some biochemical markers for obesity, inflammation, and atherosclerosis, such as ANGPTL6, hsCRP, TNF-α, IL-6, PAI-1, and triglyceride levels, and other variables after adjusting for age, BMI and gender. Multivariate logistic regression analysis was performed to evaluate the association between ANGPTL6 or MPO and the outcome of diabetes and obesity. Two sets models were created for the outcomes, Diabetes and Obesity. Both the models were adjusted for demographic factors such as age as a continuous variable and gender as a dichotomous variable. Statistical assessments were two-sided, and a *P* value <0.05 was considered significant. All analyses were performed using R Software.

**Table 1:** Demographics and characteristics of the study subjects.

	Non-Diabetes (n = 137)	Diabetes (n = 101)	p-value
Age	43.00 (31.00–53.00)	54.00 (49.00–57.00)	<0.001
Gender (Male)	52 (38.0%)	54 (53.47%)	<0.05
BMI	28.70 (24.56–33.08)	32.47 (28.52–34.78)	<0.001
Waist/Hip Ratio	0.86 (0.80–0.92)	0.94 (0.89–1.00)	<0.001
TC (mmol/L)	5.16 (4.50–5.90)	4.80 (3.85–5.80)	<0.05
HDL (mmol/L)	1.33 (1.08–1.52)	1.09 (0.89–1.29)	<0.001
LDL (mmol/L)	3.30 (2.60–3.90)	2.90 (2.07–3.90)	<0.05
TGL (mmol/L)	0.92 (0.63–1.42)	1.38 (1.06–1.90)	<0.001
FPG (mmol/L)	5.10 (4.80–5.60)	6.95 (6.00–9.38)	<0.001
HbA1c (DCCT %)	5.60 (5.30–5.90)	7.60 (6.40–8.80)	<0.001
Insulin (mIU/L)	10.23 (6.21–22.84)	16.75 (7.88–27.24)	0.08
Adiponectin (μg/mL)	4.46 (2.70–6.82)	3.02 (1.91–4.83)	<0.001
Leptin (ng/mL)	6.98 (3.85–10.17)	6.35 (4.34–10.29)	0.89
PAI-1 (ng/mL)	13.67 (11.17–18.38)	15.12 (12.81–18.65)	0.10
IL-6 (pg/mL)	16.56 (13.62–20.12)	17.22 (14.92–20.98)	0.30
TNF alpha (pg/mL)	123.45 (103.15–153.18)	128.09 (108.70–159.69)	0.46
hsCRP(μg/mL)	1.89 (0.76–4.43)	2.53 (1.24–6.65)	<0.01
Hypertension	16 (11.7%)	57 (56.4%)	<0.001
Hyperlipidemia	13 (9.5%)	34 (33.7%)	<0.001

Median (IQR) presented for continuous variables and N (%) presented for categorical variables. P values were calculated using Mann–Whitney U test for continuous variables and chi-squared test for categorical variables. BMI, body mass index; FPG, fasting plasma glucose; HbA1c, glycated hemoglobin; HDL, high-density lipoprotein; hsCRP, high sensitivity c-reactive protein; IL-6, interleukin-6; LDL, low-density lipoprotein; PAI-1, plasminogen activator inhibitor-1; TGL, triglycerides; TNF alpha, tumor necrosis factor alpha.

**Table 2:** Demographics and characteristics of the non-diabetic subjects.

	Non-Obese (n = 77)	Obese (n = 60)	p-value
Age	41.00 (31.00–52.00)	46.00 (33.75–56.00)	0.09
Gender (Male)	28 (36.4%)	24 (40.0%)	0.79
BMI	24.71 (22.46–26.86)	34.02 (31.60–36.16)	<0.001
Waist/Hip Ratio	0.84 (0.79–0.89)	0.90 (0.82–0.97)	<0.01
TC (mmol/L)	5.00 (4.35–5.75)	5.20 (4.60–5.90)	0.41
HDL (mmol/L)	1.36 (1.09–1.66)	1.17 (1.01–1.44)	<0.05
LDL (mmol/L)	3.10 (2.50–3.90)	3.50 (2.80–3.85)	0.24
TGL (mmol/L)	0.81 (0.60–1.09)	1.12 (0.66–1.55)	<0.05
FPG (mmol/L)	5.07 (4.80–5.47)	5.20 (4.90–5.80)	<0.05
HbA1c (DCCT %)	5.50 (5.30–5.80)	5.70 (5.38–5.93)	0.08
Insulin (mIU/L)	7.71 (5.21–15.14)	15.43 (8.10–29.18)	<0.001
Adiponectin (μg/mL)	4.88 (3.21–7.40)	4.07 (2.45–5.57)	0.06
Leptin (ng/mL)	5.19 (2.69–7.84)	10.07 (6.79–13.51)	<0.001
PAI-1 (ng/mL)	13.05 (10.01–16.73)	15.65 (12.55–19.13)	<0.01
IL-6 (pg/mL)	16.56 (13.19–19.77)	16.52 (14.17–20.89)	0.56
TNF alpha (pg/mL)	122.25 (104.67–160.75)	125.95 (102.36–141.80)	0.54
hsCRP(μg/mL)	1.06 (0.55–3.04)	3.07 (1.69–6.53)	<0.001
Hypertension	8 (10.4%)	8 (13.3%)	0.79
Hyperlipidemia	6 (7.8%)	7 (11.7%)	0.64

Median (IQR) presented for continuous variables and N (%) presented for categorical variables. P values were calculated using Mann–Whitney U test for continuous variables and chi-squared test for categorical variables. BMI, body mass index; FPG, fasting plasma glucose; HbA1c, glycated hemoglobin; HDL, high-density lipoprotein; hsCRP, high sensitivity c-reactive protein; IL-6, interleukin-6; LDL, low-density lipoprotein; PAI-1, plasminogen activator inhibitor-1; TGL, triglycerides; TNF alpha, tumor necrosis factor alpha.

**Table 3:** Demographics and characteristics of patients with type 2 diabetes.

	Non-obese (n = 36)	Obese (n = 65)	p-value
Age	55.00 (47.75–58.00)	53.00 (49.00–57.00)	0.95
Gender (Male)	20 (55.6%)	34 (52.3%)	0.92
BMI	27.21 (25.21–28.95)	34.19 (32.67–35.72)	<0.001
Waist/Hip Ratio	0.93 (0.87–0.97)	0.95 (0.91–1.02)	0.08
TC (mmol/L)	4.50 (3.85–5.65)	4.90 (4.02–5.82)	0.47
HDL (mmol/L)	1.04 (0.90–1.21)	1.11 (0.88–1.33)	0.56
LDL (mmol/L)	2.75 (2.02–3.70)	3.05 (2.09–3.90)	0.91
TGL (mmol/L)	1.32 (0.80–1.90)	1.38 (1.10–1.88)	0.43
FPG (mmol/L)	6.70 (5.70–8.70)	7.30 (6.40–10.40)	<0.05
HbA1c (DCCT %)	6.40 (5.90–7.60)	8.15 (6.80–9.15)	<0.001
Insulin (mIU/L)	17.51 (8.95–31.03)	15.49 (7.88–26.16)	0.69
Adiponectin (μg/mL)	2.93 (1.94–3.87)	3.33 (1.91–4.98)	0.43
Leptin (ng/mL)	5.81 (3.14–8.10)	7.85 (5.36–10.88)	<0.05
PAI-1 (ng/mL)	16.37 (14.92–20.98)	13.85 (11.69–17.43)	<0.05
IL-6 (pg/mL)	18.47 (15.95–21.95)	16.54 (13.96–20.27)	0.15
TNF alpha (pg/mL)	140.41 (125.35–168.66)	117.61 (105.84–146.38)	<0.01
hsCRP(μg/mL)	1.67 (0.74–4.75)	3.59 (2.07–7.79)	<0.001
Hypertension	18 (50.0%)	39 (60.0%)	0.45
Hyperlipidemia	13 (36.1%)	21 (32.3%)	0.87

Median (IQR) presented for continuous variables and N (%) presented for categorical variables. P values were calculated using Mann–Whitney U test for continuous variables and chi-squared test for categorical variables. BMI, body mass index; FPG, fasting plasma glucose; HbA1c, glycated hemoglobin; HDL, high-density lipoprotein; hsCRP, high sensitivity c-reactive protein; IL-6, interleukin-6; LDL, low-density lipoprotein; PAI-1, plasminogen activator inhibitor-1; TGL, triglycerides; TNF alpha, tumor necrosis factor alpha.

## Results

### Characteristics of the study population

Table 1 shows the demographic and clinical characteristics of our study population. Patients with T2D had significantly higher age, BMI, waist/hip ratio, triglyceride (TGL), fasting blood glucose (FBG) and HbA1c but lower levels of adiponectin, total cholesterol (TC), low-density lipoprotein (LDL), and high-density lipoprotein (HDL) than normal participants (P value <0.05). Furthermore, patients with T2D had significantly higher plasma high-sensitivity C-reactive protein (hsCRP), hypertension, and hyperlipidemia. Tables 2 and 3 show the characteristics of obese patients with or without T2D. Obese participants were more likely to have a significantly higher BMI, FPG, leptin, and hsCRP levels irrespective of diabetes status than non-obese participants. In patients without T2D, obese participants had significantly higher waist/hip ratio, triglyceride (TGL), insulin, and PAI-1, but lower high-density lipoprotein (HDL) levels than non-obese participants. Meanwhile, in patients with T2D, obese patients had significantly higher glycated hemoglobin (HbA1c) levels but lower PAI-1 and TNF-α levels than non-obese patients.

### Differential expression levels of ANGPTL6 and MPO in patients with T2D or obesity

**Figure 1:**
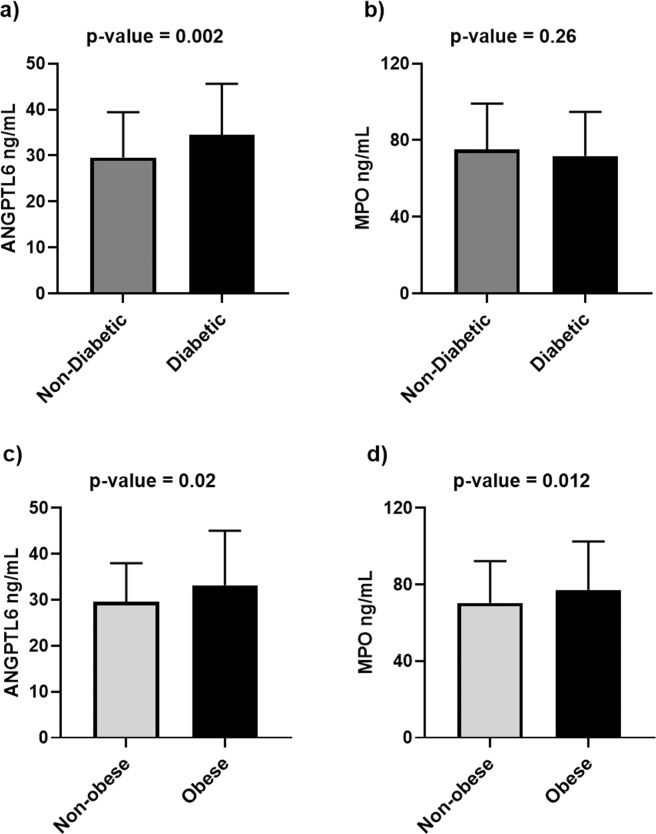
Plasma levels of ANGPTL6 and MPO in the population based on their diabetes or obesity status. (**a**) Plasma levels of ANGPTL6 in non-diabetic and diabetic subjects. (**b**) Plasma levels of MPO in non-diabetic and diabetic subjects. (**c**) Plasma levels of ANGPTL6 in non-obese and obese subjects. (**d**) Plasma levels of MPO in non-obese and obese subjects. Data are presented as mean ± SD. *P* values were determined using student’s t-test.

As shown in Fig. 1, patients with T2D had significantly elevated plasma ANGPTL6 levels (34.45 ± 11.16 ng/mL) compared to non-diabetic participants (29.57 ± 9.86 ng/mL) (Fig. 1a). By contrast, the MPO plasma levels did not differ between patients with T2D and healthy participants (Fig. 1b). When the population was stratified based on obesity status, both the plasma ANGPTL6 and MPO levels were significantly higher in obese participants (ANGPTL6 level: 33.19 ± 11.89 ng/mL and MPO level: 77.29 ± 25.22 ng/mL) than in non-obese controls (ANGPTL6 level: 29.58 ± 8.4 ng/mL and MPO level: 70.48 ± 21.76 ng/mL) (Fig. 1c,d).

### Increased levels of ANGPTL6 and MPO in obese participants with T2D

**Figure 2:**
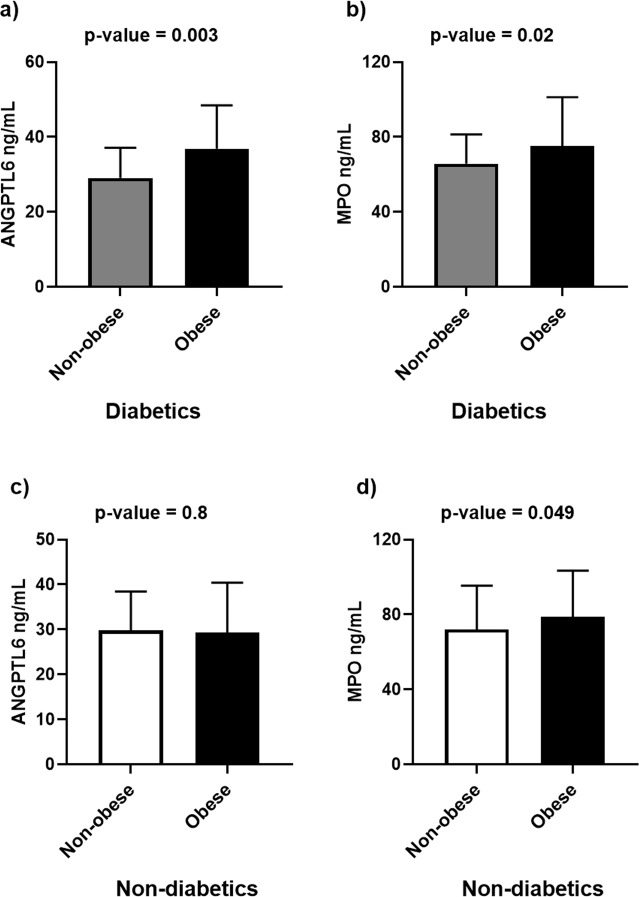
Plasma levels of ANGPTL6 and MPO in diabetic patients and non-diabetic subjects based on obesity status. (**a**) Plasma levels of ANGPTL6 in non-obese and obese diabetic patients. (**b**) Plasma levels of MPO in non-obese and obese diabetic patients. (**c**) Plasma levels of ANGPTL6 in non-obese and obese non-diabetic subjects. (**d**) Plasma levels of MPO in non-obese and obese non-diabetic subjects. Data are presented as mean ± SD. *P* values were determined using student’s t-test.

In patients with T2D, both the plasma ANGPTL6 and MPO levels were significantly higher in obese participants than in non-obese participants (ANGPTL6 level: 36.86 ± 11.55 ng/mL vs. 29.05 ± 8.08 ng/mL; P value = 0.003 and MPO level: 75.28 ± 25.94 ng/mL vs. 65.71 ± 15.64 ng/mL; P value = 0.042) (Fig. 2a,b). In contrast, in patients without diabetes, the ANGPTL6 plasma levels did not differ between obese and non-obese participants (Fig. 2c). However, the MPO plasma levels were significantly higher in the obese non-diabetic participants (78.76 ± 24.73 ng/mL) than in non-obese participants (72.08 ± 23.29 ng/mL) (Fig. 2d).

### Correlation between levels of MPO and ANGPTL6 as well as some metabolic markers

We assessed the correlation between MPO and various markers in the non-diabetic population (Table 4). The plasma MPO levels were positively associated with ANGPTL6 (r = 0.17; P < 0.05), TGL (r = 0.16; P < 0.05), TNF alpha (r = 0.19; P < 0.05), hsCRP (r = 0.15; P < 0.05), IL-6 (r = 0.22; P < 0.01), and PAI-1 levels (r = 0.21; P < 0.05) after adjusting for age and BMI. When the analysis was performed after adjusting for age and gender, this association was still observed with TNF alpha (r = 0.22; P < 0.05), hsCRP (r = 0.25; P < 0.01), IL-6 (r = 0.30; P < 0.01), and PAI-1 levels (r = 0.22; P < 0.05) but was lost for ANGPTL6 and TGL.

**Table 4:** Partial correlation between MPO plasma levels and biochemical markers for obesity, inflammation, and atherosclerosis.

Biochemical Marker	Correlation (r) (Adjusted for Age and BMI)	*P-value*	Correlation (r) (Adjusted for Age and Gender)	*P-value*
ANGPTL6	0.17	<0.05	0.11	0.27
BMI	0.20^#^	<0.01	0.19	<0.05
TGL	0.16	<0.05	0.042	0.64
TNF alpha	0.19	<0.05	0.22	<0.05
HsCRP	0.15	<0.05	0.25	<0.01
IL-6	0.22	<0.01	0.30	<0.01
PAI-1	0.21	<0.05	0.22	<0.05

^#^Adjusted for Age.

### Association between ANGPTL6 or MPO levels and T2D and obesity outcomes

Multivariate logistic regression analysis was performed to ascertain the association between MPO, ANGPTL6 and the outcome of T2D and obesity. ANGPTL6 was associated with an increased risk of T2D (adjusted OR [95% CI] = 1.04 [1.01–1.08]) and obesity (adjusted OR [95% CI]= 1.03 [1.01–1.06]). The association between MPO and T2D and obesity was not statistically significant (Supplementary Table S1).

## Discussion

The present study aimed to determine the plasma ANGPTL6 and MPO levels in both obese and diabetic individuals to validate their role in these conditions and to assess any association between MPO levels and the markers of obesity, inflammation, and atherosclerosis. Although the MPO level is upregulated in obese individuals irrespective of the diabetes status, the ANGPTL6 level is elevated in diabetic patients regardless of their BMI, which reflects the unique physiological function of each of these markers and emphasizes the need for comprehensive studies to better understand these functions and utilize them for diagnosis and therapeutic purposes.

Interestingly, our findings indicated that patients with T2D had higher plasma ANGPTL6 levels, but not MPO levels, than normal participants. Such finding was in accordance with that of two other studies conducted in Germany^29,34^ on patients with T2D, with one study showing a positive correlation between ANGPTL6 and fasting blood glucose. Similarly, serum ANGPTL6 levels were significantly higher in Asian participants who had impaired glucose tolerance than in healthy controls^31^. This finding was also observed in patients with gestational diabetes^35^. In contrast, in one study about obesity (BMI > 40 kg/m^2^), no difference was observed in terms of ANGPTL6 levels between patients with T2D and control participants^30^. This discrepancy may be attributed to the small number of diabetic patients enrolled in the study. An animal knockout model provided further evidence highlighting the association between ANGPTL6 levels and diabetes, where the targeted disruption of the *ANGPTL6* gene led to obesity and insulin resistance in mice^28^. Similarly, in previous studies, an association between other ANGPTLs and T2D is observed. For example, patients with T2D had elevated serum ANGPTL4, ANGPTL5, and ANGPTL8 levels, and a positive correlation was noted between ANGPTL4, ANGPTL5, and ANGPTL8 levels and fasting blood glucose level^23,32,36^.

The association between ANGPTL6 level and glucose metabolism is not fully understood. In DIO mice, treatment with ANGPTL6 increases adenosine monophosphate-activated protein kinase activity, which can ultimately improve insulin signaling in the skeletal muscles. Similarly, treatment of C2C12 myoblasts with ANGPTL6 can also enhance the insulin signaling pathways^37^. Another study on hepatocytes has shown that ANGPTL6 can reduce liver gluconeogenesis since it can decrease the expression of glucose-6-phosphatase via reducing the transcriptional activity of FoxO1 that results from the activation of PI3K/Akt signaling cascades^38^. Furthermore, another study has shown an increase in both hepatic and circulating ANGPTL6 levels in an obese diabetic mouse model with liver-specific deletion of glucose-6-phosphatase^39^. Taken together, our data and previous findings propose that the increase in ANGPTL6 levels in diabetes is a compensation mechanism in response to insulin resistance. In fact, a recent study has indicated that the increase in serum ANGPTL6 levels may be a predictor of metabolic syndrome^40^. In this prospective cohort study, serum ANGPTL6 levels in 221 participants without metabolic syndrome were assessed at baseline and were then followed-up after an average of 3 years. ANGPTL6 levels significantly increased before the development of metabolic syndrome and its complications; therefore, measuring ANGPTL6 levels can significantly improve the prediction of metabolic syndrome.

Our findings showing that the MPO levels did not differ between type 2 diabetic patients and control participants were in accordance with the findings of three clinical studies^41–43^. Nonetheless, the role of MPO in diabetes is still ambiguous as the findings are contrasting. For example, other clinical studies have shown elevated MPO levels in individuals with T2D^44–48^. Animal studies have shown a higher MPO activity in the vessels of diabetic Zucker rats than in non-diabetic rats^49^ and that mice deficient in MPO had improved insulin signaling and were resistant to diet-induced obesity^13^. In addition to the differences in the methodology in these studies, variations in MPO levels have been reported in a variety of conditions, such as cancer, impaired leukocyte function in diabetes, cardiovascular diseases, endothelial dysfunctions, and pregnancy (based on glycemic control) and in patients taking fenofibrate drugs^50–55^.

Inflammation plays a pathogenic role in the development of T2D. Our findings showing the elevated levels of hsCRP in patients with T2D are in accordance with those of previous studies^56–59^. It was also not surprising to have significantly higher numbers of T2D patients who have hypertension or hyperlipidemia compared with non-diabetic participants (Table 1). Obesity is associated with chronic inflammation due to the infiltration of macrophages in the adipose tissue. When a higher number of macrophages is infiltrated, a higher number of pro-inflammatory cytokines and acute-phase molecules is produced by the visceral fat^60^. A constant increase in inflammatory markers contributes to the development of chronic inflammation, atherosclerosis, and macrovascular disease in obese and diabetic patients^61–63^. Understanding the function of these inflammatory molecules can reveal the molecular association between obesity and metabolic complications.

Although ANGPTL6 is known to play a role in increasing energy expenditure and protecting against diet-induced obesity in mice^28^, its role in obesity among humans has rarely been assessed. After classifying patients with T2D according to obesity status, our findings showed that both plasma ANGPTL6 and MPO levels were significantly higher in obese than in non-obese diabetic patients. Interestingly, one study has reported that patients with metabolic syndrome who had higher waist circumference and BMI had higher serum ANGPTL6 levels than healthy controls^31^. However, considering the lack of sample segmentation based on obesity status, a clear conclusion cannot be obtained based on such findings. In this study, the high ANGPTL6 levels in patients with T2D may either indicate that this protein has a protective role against obesity, as highlighted in a previous animal study^28^, or that obesity may induce ANGPTL6 resistance in humans, as previously reported in a study about leptin^64^. Interestingly, other ANGPTLs have also been associated with obesity. For instance, serum ANGPTL4 levels were significantly higher in obese patients with impaired glucose tolerance than in lean healthy participants, and a positive correlation was observed between serum ANGPTL4 levels as well as BMI, waist circumference, fat mass, and triglyceride levels^25^. Furthermore among non-diabetic participants, serum ANGPTL3, ANGPTL4, and ANGPTL8 levels were higher in obese than in non-obese participants^23^.

By contrast, only one study has assessed the correlation between obesity and BMI with MPO levels in diabetic patients^47^ and has shown that obese diabetic patients had higher MPO levels than normal weight or underweight diabetic patients. However, the same pattern for elevated MPO levels was observed in normal participants as they become obese. This result is expected as several studies have shown that circulating MPO levels were more likely to be higher in obese participants, including children, than in normal weight participants^11,12,47,65,66^. The present data showed that MPO levels are elevated in obesity irrespective of diabetes status. Interestingly, after classifying healthy participants (non-diabetics) according to obesity status in this study, the plasma ANGPTL6 levels did not significantly differ between obese and non-obese participants, indicating that it may be dependent on the diabetes status. This result is in accordance with the findings of another study conducted in Czech Republic^30^.

Finally, a significant positive correlation was observed between MPO level and other biochemical markers, such as ANGPTL6, triglyceride, BMI, TNF-α, hsCRP, IL-6, and PAI-1. Furthermore, ANGPTL6 was associated with an increased risk of T2D and obesity. In relation to these findings, previous studies have reported an association between MPO as well as some pro-inflammatory markers and insulin resistance^12,16,55,67–69^. However, no study has reported any association between MPO and ANGPTL6. In addition, the inhibition of MPO was found useful in preventing the production of pro-oxidants and insulin resistance; therefore, it can be a potential treatment for obesity and insulin resistance. However, whether reducing the expression of MPO or just inhibiting its activity is sufficient remains to be fully elucidated.

The present study had several limitations that must be discussed. First, the mechanism of ANGPTL6 and MPO in obesity or T2D was not identified due to the cross-sectional design of the study. Thus, further longitudinal studies about the causal relationship between ANGPTL6 and MPO in T2D and obesity must be conducted. Second, our study did not include data on diet and lifestyle changes in obese or T2D patients, thus making it difficult to evaluate the effect of confounding variables. Finally, the possible effects of anti-diabetic medications on ANGPTL6 and MPO levels were not assessed, even though fenofibrates are known to lower MPO levels.

## Conclusions

In this study, the role of MPO and ANGPTL6 on diabetes and obesity was assessed, and results showed that although both markers are elevated in obese participants, diabetes status can affect their physiological function. Thus, future studies should evaluate the beneficial effects of targeting MPO and ANGPTL6 simultaneously for the treatment of both obesity and T2D by inhibiting the activity of MPO and overexpression of ANGPTL6. In addition, the two markers may be affected at an earlier time before the development of the disease; therefore, it could be targeted using a preventative approach.

## Supplementary information

Supplementary information.
