# Supplementary material for: Investigating the Role of Myeloperoxidase and Angiopoietin-like Protein 6 in Obesity and Diabetes
Source: Sci Rep. 2020 Apr 10;10:6170. doi: 10.1038/s41598-020-63149-7 (PMC7148302; doi:10.1038/s41598-020-63149-7)
Supplement: Supplementary file 1 — Supplementary information. [file 41598_2020_63149_MOESM1_ESM.pdf]

Investigating the Role of Myeloperoxidase and Angiopoietin-like Protein 6 in Obesity and Diabetes

Mohammad G. Qaddoumi [1,2], Muath Alanbaei [3], Maha M. Hammad [1], Irina Al-Khairi [1], Preethi Cherian [1], Arshad Channanath[4], Thangavel Alphonse Thanaraj[4], Fahd Al-Mulla [4], Mohamed Abu-Farha [1*], and Jehad Abubaker [1*]

1 Biochemistry and Molecular Biology, Dasman Diabetes Institute, Kuwait City, Kuwait.
2 Pharmacology and Therapeutics Department, Faculty of Pharmacy, Kuwait University, Kuwait City, Kuwait.
3 Department of Medicine, Faculty of Medicine, Kuwait University, Kuwait City, Kuwait.
4 Functional Genomic Unit, Dasman Diabetes Institute, 15462 Kuwait City, Kuwait;

* Correspondence: jehad.abubakr@dasmaninstitute.org; mohamed.abufarha@dasmaninstitute.org; Tel.: +965 2224-2999

Supplementary Table S1: Association between circulating ANGPTL6 or MPO levels and the outcomes of diabetes and obesity using multivariate logistic regression

	T2D AOR [95%CI]	p-value	Obesity AOR [95%CI]	p-value
ANGPTL6 (ng/mL)	1.04 [1.01-1.08]	0.005	1.03[1.01-1.06]	0.023
[redacted]				
MPO (ng/mL)	0.99 [0.98-1.01]	0.694	1.01[0.99-1.02]	0.065
[redacted]				

ANGPTL6: Angiopoietin-like protein 6; AOR: Adjusted odds ratio (adjusted for age and gender); MPO: Myeloperoxidase
